# Energy expenditures & physical activity in rats with chronic suboptimal nutrition

**DOI:** 10.1186/1743-7075-3-11

**Published:** 2006-01-31

**Authors:** Russell Rising, Fima Lifshitz

**Affiliations:** 1 EMTAC Inc., 651 Vanderbilt St, #6J Brooklyn, NY 11218, USA; 2 Sansum Medical Research Institute, 2219 Bath Street, Santa Barbara, CA 93105, USA

## Abstract

**Background:**

Sub-optimally nourished rats show reduced growth, biochemical and physiological changes. However, no one has assessed metabolic rate adaptations in rats subjected to chronic suboptimal nutrition (CSN). In this study energy expenditure (EE; kcal/100 g body weight) and physical activity (PA; oscillations in weight/min/kg body weight) were assessed in rats subjected to three levels of CSN.

**Results:**

Body weight gain was diminished (76.7 ± 12.0 and 61.6 ± 11.0 g) in rats fed 70 and 60% of the ad-libitum fed controls which gained more weight (148.5 ± 32.3 g). The rats fed 80% gained weight similarly to controls (136.3 ± 10.5 g). Percent Fat-free body mass was reduced (143.8 ± 8.7 and 142.0 ± 7.6 g) in rats fed 70 and 60% of ad-libitum, but not in those fed 80% (200.8 ± 17.5 g) as compared with controls (201.6 ± 33.4 g). Body fat (g) decreased in rats fed 80% (19.7 ± 5.3), 70% (15.3 ± 3.5) and 60% (9.6 ± 2.7) of ad-libitum in comparison to controls (26.0 ± 6.7). EE and PA were also altered by CSN. The control rats increased their EE and PA during the dark periods by 1.4 ± 0.8 and 1.7 ± 1.1 respectively, as compared with light the period; whereas CSN rats fed 80 and 70% of ad-libitum energy intake had reduced EE and PA during the dark periods as compared with the light period EE(7.5 ± 1.4 and 7.8 ± 0.6 vs. 9.0 ± 1.2 and 9.7 ± 0.8; p < 0.05, respectively), PA(3.1 ± 0.8 and 1.6 ± 0.4 vs. 4.1 ± 0.9 and 2.4 ± 0.4; p < 0.05) and RQ (0.87 ± 0.04 and 0.85 ± 0.5; vs. 0.95 ± 0.03 and 0.91 ± 0.05 p < 0.05). In contrast, both light (7.1 ± 1.4) and dark period (6.2 ± 1.0) EE and PA (3.4 ± 0.9 and 2.5 ± 0.5 respectively) were reduced in rats fed 60% of ad-libitum energy intake.

**Conclusion:**

CSN rats adapt to mild energy restriction by reducing body fat, EE and PA mainly during the dark period while growth proceeds and lean body mass is preserved. At higher levels of energy restrictions there is decreased growth, body fat and lean mass. Moreover EE and PA are also reduced during both light and dark periods.

## Background

Suboptimal nutrition in children, due to a chronic reduction of energy/nutrient intake over a long period of time, causes a deceleration of growth accompanied by inadequate weight gain [1]. The deceleration of growth may be an adaptation mechanism to suboptimal nutrient intake that results in short stature [2,3]. Nutritional growth retardation is a hallmark of insufficient nutrient intake world wide [4,5]. In affluent societies nutritional growth retardation of non-organic origin is often associated with health beliefs without disturbed psychosocial function [6]. This syndrome is also associated with a decrease in erythrocyte sodium-potassium ATP-ase activity without any other biochemical alterations [7].

The biochemical and hormonal changes associated with chronic suboptimal nutrition have been studied utilizing a rodent model [8]. Sodium-potassium ATPase activity was reduced in suboptimally fed rats which received 60% of ad-libitum energy intake [8]. However, body weight gain was preserved in suboptimally fed rats treated with recombinant human growth hormone [9,10]. Furthermore, simultaneous restriction of both energy and zinc did not enhance the detrimental effects of chronic suboptimal nutrition on growth [11]. Moreover, substitution of fat for carbohydrates leads to greater body weight gain through a reduction of energy expenditure and possibly decreasing leptin secretion [12]. Other changes due to chronic suboptimal nutrition include a reduction of liver weight with an increase in percent total polyunsaturates, n-6 polyunsaturates and total unsaturates in mitochondrial lipids [13]. Suboptimal nutrition also reduces mandibular and femur bone growth with a greater effect on the femur bone [14,15]. Furthermore, insulin, glucose and leptin levels were reduced in Wistar rats subjected to a 35% reduction of caloric intake for five months [16]. Finally, rats suboptimally fed for three weeks showed reduced T-cell numbers in the thymus [17]. All these studies suggest that minor biochemical and physiological changes do occur during chronic suboptimal nutrition. However, there are no data on the changes in the continuous 24-hour metabolic and physical activity profiles along with related changes in body composition of rodents undergoing various degrees of long term chronic suboptimal nutrition. This report deals with the alterations with the metabolic profile which contributes to preservation of growth, body weight and fat-free mass with mild restriction of energy intake and with the inability of the rats to maintain growth with greater energy restriction despite reduced energy expenditures.

## Materials and methods

Forty-four pre-pubertal four-week old male Sprague-Dawley rats were studied at the Miami Children's Hospital Research Institute in Miami Florida. All experimental protocols were reviewed and approved by the Animal Care and Use Committee of that institution. The animals were individually housed in wire-bottom stainless steel cages avoiding coprophagia. The light/dark cycle maintained within the rodent facility was 12/12 hours respectively beginning at 7:00 AM. The rats were divided into four groups. Twenty-two were ad-libitum fed controls while 8, 7 and 7 rats, respectively, were pair-fed at 80, 70 and 60% of the amount of energy consumed by their ad-libitum fed counterparts. They were fed a balanced purified diet containing 1:1 carbohydrate: fat (Purina Mills Test Diets, Richmond, IN) providing 3.94 kcal/g. The amount of energy from carbohydrate, fat and protein of this diet was 38.3, 38.3 and 23.4%, respectively, for every gram fed. This diet was adjusted for total energy according to the following [18]:

1) Diet A – 100% of energy and all nutrients, fed ad-libitum

2) Diet B – 80% of energy and 100% of nutrients, pair-fed with rats in group A

3) Diet C – 70% of energy and 100% of all the nutrients, pair-fed with rats in group A

4) Diet D – 60% of energy and 100% of all nutrients, pair-fed with rats in group A

All rats were fed their experimental diets for four-weeks prior to the start of metabolic measurements. Purified diets were used in order to precisely control the amount of nutrients and to eliminate the variability often associated with commercial rat chows. The composition of the experimental diets is shown in Table 1. The control diet (Diet A) was formulated to contain all nutrients, including vitamins and minerals, necessary for normal growth in rats as defined by the National Research Council [18]. The dietary guidelines for rodents were based on rats consuming approximately 60 calories (15 g) of commercial rat chow per day. The formulation of each diet and the related modifications to the energy levels for the restricted diets (Diets B, C and D) were based on consumption of similar diets in three previous studies of suboptimal nutrition in rats [8-10].

**Table 1:** Composition of experimental diets

Energy Content (%)^1^	100	80	70	60
Casein (92% purity)	24.8	24.8	24.8	24.8
DL-Methionine	0.22	0.30	0.34	0.37
Sucrose	15.00	15.00	15.00	15.00
Dextrin	22.70	22.70	22.70	22.70
Lard	8.37	8.37	8.37	8.37
Corn Oil	8.37	8.37	8.37	8.37
Choline bitartrate	0.15	0.22	0.22	0.22
Vitamin mix	0.74	0.97	1.12	1.26
Mineral mix	2.60	3.27	3.79	4.31
Alpha cellulose	17.09	16.04	15.33	14.64

^1 ^= The amount of each ingredient is the percentage of the total diet

All nutrients, except energy, were concentrated to compensate for the energy restriction of each diet. The percentages of metabolisable energy from fat, carbohydrate and protein were adapted from McCargar et al [19]. Because the rats were provided excess protein over the minimal requirement (15% for growth, maintenance and breeding) in the ad-libitum diet (Table 1), it was not necessary to adjust crude protein content for formulation of the restricted diets. All control and restricted rats were consuming approximately 23 and 16% protein, respectively. However, micronutrient levels were maintained at adequate concentrations by increasing their levels in the restrictive diets. The percentage of alpha cellulose was reduced to accommodate the increased concentrations of micronutrient mixes (Table 1). Rats consumed deionized water ad-libitum. Furthermore, this water was used for food preparation. This eliminated any effect of additional micronutrients that might be contained in tap water.

Food fed to the restricted rats was calculated based on the amount of food consumed by the respective pair mates in the ad-libitum fed groups. For example, the amount of food given to the rats paired at 60% of energy intake was calculated as follows: [(food consumed by the ad-libitum fed pair-mate during the previous day/weight of this rat in the previous day) × (0.6) × (current weight of the rat for which the food was estimated)]. Daily body weights were recorded during the light period beginning at 8:00 AM prior to feeding of the experimental diets. Weighing and feeding the rats took approximately two hours each morning. Caloric efficiency (kcal/g body weight gain) was also calculated daily by multiplying food intake (g) by the caloric content of the diet and dividing this result by daily body weight gain (g). Delta body weight gain was calculated by subtracting the body weight of the rat the day of the metabolic test from that of the first day of the experiment. The daily body gain was calculated by taking body weight gain and dividing by the number of days between the start of the experiment and the day of the metabolic test. After the completion of the metabolic tests all rats were anesthetized with Nembutal (30–50 g/kg body weight) and killed by cardiac puncture in the morning at the same time of recording of body weight. Carcasses were frozen at -20 degrees C for measurements of body composition using Folch's analysis method for fat extraction [20]. All analytical measurements were conducted within one week after freezing of samples.

### Metabolic assessment

Metabolic measurements for each rat started after completion of four-weeks of feeding. On the day of the metabolic study each rat was weighed and had total energy expenditure (EE; kcal/100 g body weight), respiratory quotient (RQ;VCO_2_/VO_2_) and an index of physical activity (PA; oscillations/min/kg body weight) measured in the rodent Enhanced Metabolic Testing Activity Chamber (EMTAC). The main analytical unit of this instrument was developed to be suitable for various applications in both humans and animals for comprehensive measurements of energy expenditure and physical activity [[11,21], and [22]]. For this study, the EMTAC was retrofitted with a 72 liter Plexiglas rodent enclosure. Measurements of total energy expenditure and the index of physical activity consisted of first placing the rat, along with the appropriate experimental diet and deionized water, in a standard Nalgene metabolic cage. This cage was inserted into the EMTAC rodent enclosure at 8:00 AM and energy expenditure determinations were done by measurement of oxygen and carbon dioxide exchange within the enclosure. The Nalgene cage was designed for large rats thus minimizing the stress of a changing environment on the rodent. The light/dark cycle was maintained at 9/12 hours respectively during metabolic measurements. This allowed three hours after the dark period for data collection in regards to food intake, body weight and calculation of the next days food needs for all the rats in the study. Moreover, enclosure cleaning and instrument calibrations were conducted during this time.

The PA index was obtained by placing the entire rodent enclosure on a balance that was connected to the EMTAC unit's computer. The oscillations in weight (g), generated by movements of the rat, were read from the balance and utilized to calculate an index of physical activity expressed as oscillations in weight (g)/minute/kg body weight of the rat. For these calculations the software calculated the body weight of the rat in kilograms. The formulas used to calculate energy expenditure and physical activity index using the EMTAC have been validated and described previously [11,21,22]. Total energy expenditure data was expressed per 100 g of body weight to factor out the effects of body weight changes on metabolic rate. Metabolic measurements were conducted for a total of 21 hours and EE results extrapolated to 24 hours.

Due to the limited number of days available for metabolic measurements only 12, out of the 22 ad-libitum fed rats, were randomly selected for metabolic measurements. Metabolic measurements performed on control rats of similar weight in three previous suboptimal nutrition experiments had minimal variations in EE and PA [[9,10], and [11]]. Furthermore, there were no differences in body weight at the end of the experimental period between the ad-libitum fed rats that had metabolic measurements (271 ± 18.3 g) and those that did not (272 ± 26.8 g).

### Body composition analysis

Body composition analysis included total body water content (TBW), fat-free mass (FFM) and body fat (BF) determinations. This procedure comprised three different steps: 1) carcass homogenization, 2) water content and dry weight determinations and 3) fat content determination by fat extraction. The first step consisted of autoclaving each animal carcass for one hour at 15.3 psi and 120 degrees C in order to facilitate carcass homogenization. Each carcass was individually placed in a large beaker with covered tops with a known amount of distilled water. Each carcass was allowed to cool off overnight and then homogenized using a PowerGen700 blender. Triplicate aliquots of the homogenate were frozen at -20 degrees C for subsequent analysis. The chemical analysis of each homogenized carcass was carried out in triplicate and mean values of these triplicate samples were taken as ultimate values of total body water (TBW) and body fat (BF) content. The second step consisted of drying samples overnight at -380 mm Hg at 40 degrees C in a vacuum oven to determine water content. The difference between petri dish weight before and after overnight water extraction was considered as the dry weight of the carcass sample.

The third step consisted of determining BF content by a modified Folch's method [20] for fat extraction. All samples were analyzed following this technique's protocol which comprised of two different procedures. In the first procedure, lipids were extracted from the homogenate by adding a 2:1 methanol-chloroform mixture. Each sample was separately filtered and a 5-fold volume of distilled water was added to separate lipids from non-lipid substances. This mixture was centrifuged for 15 minutes at 3000 rpm at three degrees C, producing three separated layers. The clear upper layer contained a mixture of methanol and water. The fluffy middle layer contained non-lipid substances and the clear lower layer contained a mixture of tissue lipids and chloroform. This bottom layer containing lipids and chloroform was isolated by removing the upper and the middle layers by vacuum aspiration. During the second procedure, all samples containing only the remaining bottom layer were dried overnight at -380 mm Hg at 40 degrees C in a vacuum oven, thus allowing chloroform evaporation and subsequent lipid separation. The difference between the tubes weight before and after fat extraction was taken as grams of fat content [23]. Fat-free mass was calculated by subtracting BF percentage from total body mass and expressed as grams of fat-free mass.

### Statistical analysis

One way ANOVA utilizing Least Significant Difference (LSD) was utilized to determine the differences for EE, RQ, PA, food intake, caloric efficiency, initial and final body weights, delta body weight gain, fat-free and fat mass and percent total body water between the ad-libitum and each of the energy restricted groups. One way ANOVA with LSD was also utilized to determine differences in EE, RQ and PA between the ad-libitum and restricted energy groups during both the light and dark periods. Differences of all metabolic parameters (EE, RQ and PA) within each dietary treatment group between the light and dark periods were analyzed utilizing paired t-test. All data are presented as mean ± standard deviation (SD) unless otherwise noted.

## Results

**Figure 1:**
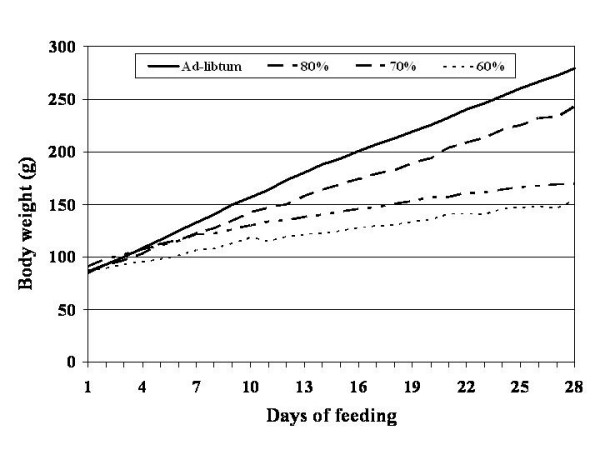
Daily body weight profile for those rats fed ad-libitum and at 80, 70 and 60% of ad-libitum energy intake for 28 days. * = p < 0.05 in comparison to ad-libitum fed controls utilizing ANOVA with LSD.

All rats appeared healthy and gained weight throughout the experimental period (Table 2). Despite a dietary energy restriction of up to 60% of ad-libitum intake, all rats gained some body weight each day of the experiment (Figure 1). However, restricted rats achieved a final lower body weight as compared with the ad-libitum fed controls (Table 2). None of the rats lost weight on any given day throughout the experimental period.

**Table 2:** Food intake, caloric efficiency, body weight and composition of rats after four weeks on the experimental diets

Level (%)	Food intake (g/100 g BW)	Caloric efficiency (kcal/g)	Initial body weight (g)	Final body Weight (g)	Delta body weight gain (g)	Fat-free mass (g)	Fat-mass (g)	Total body water (%)
100	34.8 ± 7.1	21.8 ± 1.6	80.6 ± 8.4	227.6 ± 38.3	148.5 ± 32.3	201.6 ± 33.4	26.0 ± 6.7	62.0 ± 5.4
80	27.9 ± 5.7*	23.1 ± 2.7	84.3 ± 9.0	220.5 ± 17.5	136.3 ± 10.5	200.8 ± 17.5	19.7 ± 5.3*	66.7 ± 5.1
70	17.7 ± 1.7*	19.9 ± 1.9	82.4 ± 2.9	159.1 ± 10.0*	76.7 ± 12.0*	143.8 ± 8.7*	15.3 ± 3.5*	61.7 ± 6.9
60	16.0 ± 2.4*	27.3 ± 7.8*	90.0 ± 9.4	151.6 ± 7.7*	61.6 ± 11.0*	142.0 ± 7.6*	9.6 ± 2.7*	67.3 ± 3.3*

* = p < 0.05 in comparison to ad-libitum fed controls utilizing ANOVA with LSD.

Mean daily food intake across the four week experiment was lower for those rats fed 80, 70 and 60% of ad-libitum intake (p < 0.05) in comparison to the full fed controls (Table 2). However, caloric efficiency was increased significantly (p < 0.05) only in those rats fed at 60% of ad-libitum energy intake when compared to ad-libitum fed controls (Table 2). Delta body weight gain was reduced (p < 0.05) in those rats fed at 70 and 60% of ad-libitum energy intake. However, rats fed 80% of ad-libitum energy intake gained weight at a level similar to controls (Table 2). This represented an 8.2, 48.3 and 58.5% reduction of total body weight gain over the course of the experiment as compared with controls, for those rats fed 80, 70 and 60% of ad-libitum energy intake respectively,. Daily body weight gain also showed significant (p < 0.05) reductions which were apparent in those rats fed at 70% of ad-libitum intake and were more sever**e **in those fed 60% (Table 2). This represented a 49 and 58% reduction in daily body weight gain for those rats fed 70 and 60% of ad-libitum energy intake, respectively, as compared with ad-libitum fed rats.

There were changes in body composition associated with chronic suboptimal nutrition. Fat-free and fat mass decreased (p < 0.05) in those rats fed at 70 and 60% of ad-libitum energy intake in comparison to ad-libitum fed controls. The fat mass (p < 0.05) also decreased in those rats fed at 80% ad-libitum energy intake but the fat-free mass was preserved (Table 2). Moreover, there was a slight increase in total body water beginning with rats fed only 80% of ad-libitum energy intake. Significant increases (p < 0.05) occurred in those rats fed at 60% of ad-libitum energy intake (Table 2).

**Figure 2:**
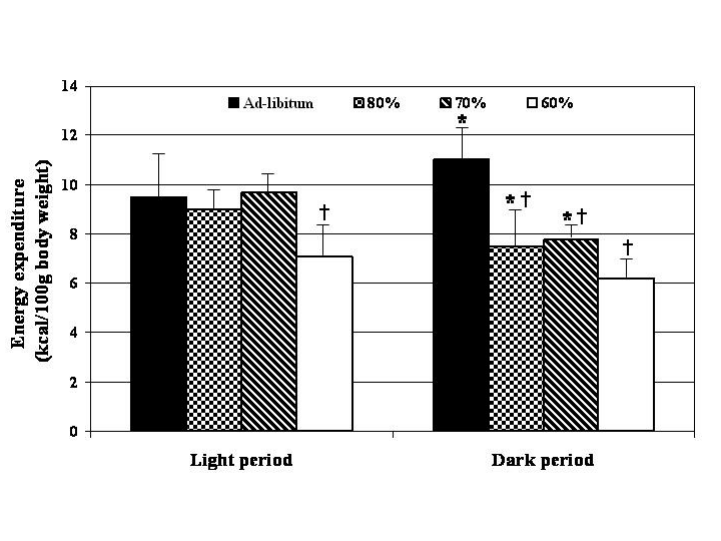
Comparison of energy expenditure between the light and dark periods. * = p < 0.05 between the light and dark periods within each feeding level. Significance determined by pair T- tests. † = p < 0.05 from ad-libitum fed (100%) control rats. Significance determined by ANOVA with LSD.

**Figure 3:**
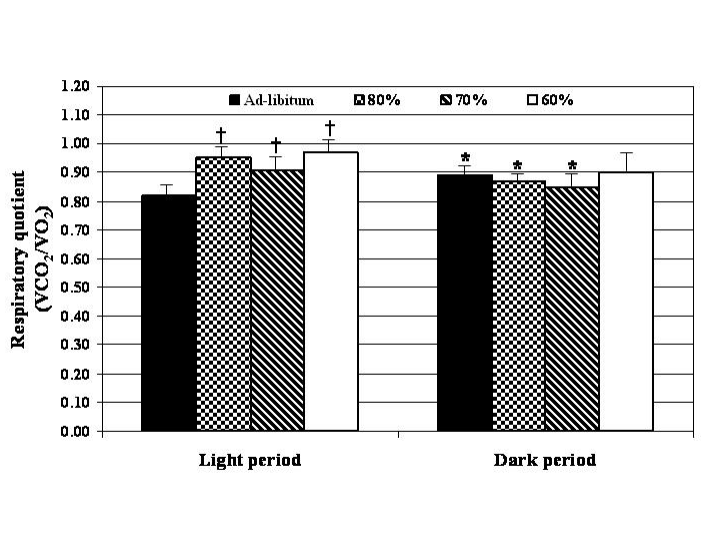
Comparison of respiratory quotient between the light and dark periods. * = p < 0.05 between the light and dark periods within each feeding level. Significance determined by pair T- tests. † = p < 0.05 from ad-libitum fed (100%) control rats. Significance determined by ANOVA with LSD.

**Figure 4:**
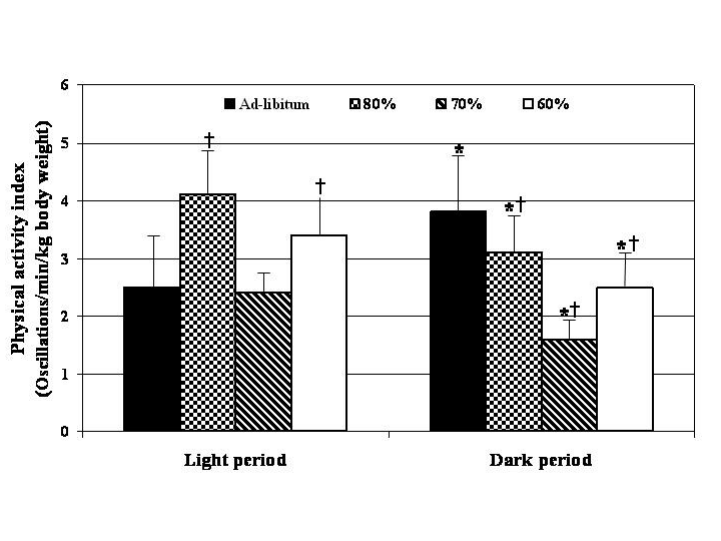
Comparison of physical activity index between the light and dark periods. * = p < 0.05 between the light and dark periods within each feeding level. Significance determined by pair T- tests. † = p < 0.05 from ad-libitum fed (100%) control rats. Significance determined by ANOVA with LSD.

The differences between the light and dark periods within each dietary treatment group for energy expenditure (kcal/100 g body weight), respiratory quotient (VCO_2_/VO_2_) and physical activity index (oscillations/min/kg body weight) are shown in Figures 2, 3, 4. In comparison to the light period, rats fed ad-libitum showed increased (p < 0.05) EE and PA during the dark period, while those rats fed at 80 and 70% of ad-libitum showed significant (p < 0.05) decreases (Figure 2 and 3). No differences in energy expenditure were detected in those rats fed at 60% of ad-libitum intake between dark and light periods. However, physical activity was significantly decreased (p < 0.05) in those rats fed at 80, 70 and 60% of ad-libitum (Figure 4). As expected, respiratory quotient increased (p < 0.05) in the ad-libitum fed controls during the dark period while those rats fed at 80 and 70% of ad-libitum showed significant (p < 0.05) decreases (Figure 3). No changes in the respiratory quotient were found between the light and dark periods in those rats fed at 60% of ad-libitum.

The data for each restricted group were also compared to the control rats within both the light and dark periods. During the light period those rats fed at 60% of ad-libitum had significantly (p < 0.05) reduced EE in comparison to the controls (Figure 2). During the dark period those rats fed 80, 70 and 60% of ad-libitum had reduced EE in comparison to control rats. In regards to the respiratory quotient (VCO_2_/VO_2_), those rats fed 80, 70 and 60% of ad-libitum intake had a greater respiratory quotient during the light period in comparison to control rats (Figure 3). The results for PA oscillations/min/kg body weight) were variable. Physical activity index (oscillations/min/kg body weight) was increased (p < 0.05) during the light period in comparison to the control rats. However, PA was decreased during the dark period when compared to control rats (Figure 4).

## Discussion

This is the first study of the daily metabolic profile in rodents subjected to various levels of chronic suboptimal nutrition. We utilized the rodent EMTAC to conduct accurate 21-hour measurements of EE and PA in rats restricted to 80, 70 or 60% of ad-libitum energy consumed by controls. This allowed us to determine the effects of chronic minor (suboptimal) levels of energy restriction on the energy expenditure during both the light and dark periods. Rats that were restricted to only 80% of their ad-libitum energy intake grew at a rate comparable to the ad-libitum fed controls. Furthermore, they preserved fat-free mass but had reduced EE and PA, along with increased RQ during the dark period. These data indicate that these rats utilize their body fat and reduced their physical activity to conserve energy for growth and lean body mass maintenance. However, there may be other effects at this level of energy restriction, not measured in this study, such as reduction of oxygen free radicals from oxidation of substrates [13] and impaired immune function [17,24] or other biochemical and hormonal changes as reported by other investigators with different models of severe malnutrition [25-27]. However, rats fed only 70% of ad-libitum energy intake had reductions in both growth and lean body mass and had a greater magnitude in the reduction of EE & PA. Rats subjected to even greater amounts of energy restriction, such as those fed 60% of ad-libitum energy intake, had greater detrimental effects such as reduced growth, loss of lean body and fat mass along with further decreases in EE and PA. Moreover, their RQ was increased during both the light and dark periods. Despite only consuming up to 60% of their ad-libitum energy intake, these rats still preserved 26% of their body weight gain as compared to ad-libitum fed controls. Furthermore, they showed increased caloric efficiency. These results suggests that over the course of the four-week experiment, some essential needs of metabolism were still being met and a minimal amount of energy was still available for growth. Under a greater restriction level, an increase in caloric efficiency may contribute to the preservation of body weight gain. However, it is not known what effects a longer period of restriction would have on these rats.

Few studies have reported any differences between the light and dark periods in regards to energy metabolism and physical activity [25-28]. Rats are normally more active during the dark period as seen in the ad-libitum fed rats in our study. This activity cycle is controlled by suprachismatic nuclei in the hypothalamus responding to afferent and efferent responses [28]. However, once energy restriction begins, as in our case a 20% reduction in energy intake, most of the changes in EE and PA appeared during the dark period. It is possible that only subtle changes such as a reduction of EE and PA, such as found in our study, is enough to maintain some body weight gain and good general health without the more severe effects of a greater caloric restriction.

There are other physiological adaptations that might contribute to the maintenance of health and body weight gain during chronic suboptimal nutrition. For example, a reduction of body temperature might be another energy conservation mechanism. Chronic caloric restriction up to 60% of ad-libitum energy intake has been found to reduce body temperature in mice [26] and rats [29]. In another study, rats subjected to three days of starvation showed a lowering of body core temperature during the light period. Furthermore, the preference ambient temperature of these starved rats was greater then the ad-libitum fed controls [30]. Since changes in EE are directly related to body temperature [31] this suggests that part of the metabolic adaptation during chronic suboptimal nutrition may be a reduction of body temperature. However, we did not measure body temperature in our study.

It is possible that other factors might also contribute to the preservation of metabolic homeostasis. For example, there are alterations in erythrocyte sodium-potassium-ATPase activity which accounts for approximately one third of the basal energy metabolism [32]. In a previous study, rats fed 60% of ad-libitum energy intake for a four-week period showed reduced sodium-potassium-ATPase activity [8]. Moreover, it is also possible that the amount of fat in the diet could affect EE. It has been reported that rats fed 3:1 and 2:1 ratios of fat to carbohydrate diets for two weeks had lower EE than those fed only a 1:1 fat to carbohydrate diet [12]. It is possible that these above changes occurred in our rats thus contributing to the preservation of body weight gain with energy restriction up to 60% of ad-libtium.

There are several potential reasons why sub optimally fed rats in our study had a higher RQ when compared to controls. For example, chronic suboptimal energy intake lasting up to 30 days might cause increased insulin sensitivity. This has been suggested as the cause of the greater RQ found in human anorexia nervosa patients [33]. Moreover, injected ghrelin increased glucose oxidation, and subsequently RQ, in normally fed rats during the dark period [34]. It is possible that chronic suboptimal nutrition in rodents for 30 days may cause hormonal changes or shifts in nutrient oxidation that might increase the RQ.

It has been reported by numerous investigators [[35,36], and [37]] that mild energy restriction will extend life span in rodents. It is possible that our rats fed 80% of ad-libitum energy intake might potentially live longer and maintain better health throughout their life span. These rats showed a significant reduction of body fat while preserving lean body mass. However, we did not study this aspect and did not continue them at restricted levels of energy intake throughout their entire life span. Other studies in rodents have found that life-spans were increased when energy intake was restricted in mice and rats up to 60% of ad-libitum intake [[35,36], and [37]]. Furthermore, other studies have found that caloric restriction started at middle age in mice still increased their average maximum lifespan by up to 20% [37]. One reason for the lengthening of the lifespan may be a reduction of the insulin/IGF signaling pathway. Reduction in the activity of this pathway may allow expression of certain genetic factors thus contributing to the lengthening of the lifespan [38]. However it is difficult to visualize an appropriate homeostasis that will allow optimal health and prolongation of life with levels of energy restriction that are associated with a degradation of lean body mass.

## Conclusion

We have demonstrated that energy restriction up to 60% of ad-libitum energy intake causes detrimental effects such as reduced lean body mass, energy expenditure and physical activity. We also found that rats only restricted to 80% of their ad-libitum energy intake preserves their fat-free mass and maintenance of normal body weight gain. It appears their main energy needs for growth were obtained by a reduction of their fat mass and physical activity during the dark period. Moreover, we found that most of the alterations in energy metabolism and physical activity occurred during the dark period.

## Abbreviations

CSN = Chronic suboptimal nutrition

EMTAC = Enhanced Metabolic Testing Activity Chamber

ANOVA = Analysis of Variance

LSD = Least Significant Difference

SD = Standard Deviation

BF = Body Fat

FFM = Fat-free mass

TBW = Total body water

BW = Body weight

EE = Energy expenditure

RQ = Respiratory quotient

PA = Physical activity

PSI = Pounds per square inch

H_2_O_2 _= Hydrogen peroxide

## Competing interests

The author(s) declare that they have no competing interests.

## Authors' contributions

Dr. Russell Rising has contributed to the design of the experiment and conducted the data analysis. Furthermore, he either participated in some of the actual data acquisition or supervised pediatric research fellows in this regard. He also assisted in the preparation of the small grants necessary for funding of this project. Finally, he also assisted in the writing and editing of this manuscript.

Dr. Fima Lifshitz contributed with ideas for the study, the design of the experiment, the preparation of the manuscript and assisted with data analysis. He also elicited the grants and funding necessary for the financial support of this study. Both authors were involved in the final editing of this manuscript.
